# Supplementary material for: Unexpectedly High Prevalence of Breakfast Skipping in Low Body-Weight Middle-Aged Men: Results of the Kanagawa Investigation of Total Checkup Data from the National Data Base-7 (KITCHEN-7)
Source: Nutrients. 2020 Dec 30;13(1):102. doi: 10.3390/nu13010102 (PMC7823689; doi:10.3390/nu13010102)
Supplement: Supplementary file 1 [file nutrients-13-00102-s001.pdf]

Supplementary Table 1. Odds ratios and 95% confidence intervals of BMI categories for breakfast skipping (BS).

BMI (kg/m^2)	≤ 16.9	17.0 - 18.9	19.0 - 20.9	21.0 - 22.9	23.0 - 24.9	25.0 - 26.9	27.0 - 28.9	29.0 - 30.9	31.0 - 32.9	≥ 33.0
Number										
Whole	16,928	86,081	182,221	224,909	184,864	105,434	50,872	22,933	10,101	8,235
Men	2,822	19,313	6,7099	126,654	125,442	75,058	35,794	15,520	6,501	5,000
Women	14,106	66,768	115,122	98,255	59,422	30,376	15,078	7,413	3,600	3,235
Crude Odds ratio										
Whole	0.86*** (0.82 - 0.90)	0.85*** (0.84 - 0.87)	0.92*** (0.91 - 0.94)	1	1.10*** (1.08 – 1.12)	1.22*** (1.20 - 1.24)	1.38*** (1.34 - 1.41)	1.49*** (1.44 - 1.54)	1.64*** (1.56 – 1.72)	1.77*** (1.68 - 1.86)
Men	1.45*** (1.33 - 1.57)	1.34*** (1.29 - 1.39)	1.13*** (1.10 - 1.15)	1	1.00 (0.98 - 1.02)	1.08*** (1.06 - 1.11)	1.22*** (1.19 - 1.26)	1.35*** (1.29 - 1.40)	1.49 (1.41 – 1.58)	1.56*** (1.46 - 1.66)
Women	1.08** (1.02 - 1.14)	1.00 (0.97 - 1.03)	1.01 (0.98 - 1.04)	1	1.07*** (1.03 – 1.10)	1.20*** (1.15 - 1.25)	1.40*** (1.33 - 1.48)	1.54*** (1.44 - 1.64)	1.77*** (1.61 – 1.93))	2.16*** (1.98 - 2.37)
Adjusted Odds ratio										
Whole	1.10*** (1.05 - 1.16)	1.01 (0.99 - 1.04)	1.01 (0.98 - 1.03)	1	1.03 (1.00 - 1.05)	1.07*** (1.04 - 1.10)	1.14*** (1.13 - 1.23)	1.18*** (1.17 - 1.31)	1.24*** (1.17 – 1.31)	1.28*** (1.21 - 1.36)
Men	1.40*** (1.28 - 1.54)	1.23*** (1.19 - 1.28)	1.07*** (1.05 - 1.10)	1	1.00 (0.98 – 1.02)	1.04** (1.01 - 1.06)	1.09*** (1.06 - 1.12)	1.11*** (1.07 - 1.16)	1.15*** (1.08 - 1.22)	1.12*** (1.04 – 1.19)
Women	1.11** (1.04 - 1.18)	1	1.05** (1.02 - 1.09)	1.11*** (1.07 -1.15)	1.20*** (1.15 -1.24)	1.27*** (1.22 - 1.33)	1.42*** (1.34 - 1.50)	1.47*** (1.37 - 1.59)	1.55*** (1.40 - 1.71)	1.71*** (1.55 - 1.88)

*P < 0.05, **P < 0.01, ***P < 0.001

Adjusted Odds ratios were calculated by controlling for age, smoking, pharmacotherapy for hypertension, pharmacotherapy for dyslipidemia, history of cardiovascular disease, moderate to heavy consumption of alcohol (≥ 46 g ethanol per day), habitual exercise, LND (late night dinner), LNS (late night snack), and sex (in the case of all subjects).

BMI, body mass index; BS, breakfast skipping.

Supplementary Table 2. Odds ratios and 95% confidence intervals of WC categories for breakfast skipping (BS).

WC (cm)	≤ 64.9	65.0 - 69.9	70.0 - 74.9	75.0 - 79.9	80.0 - 84.9	85.0 - 89.9	90.0 - 94.9	95.0 - 99.9	100.0 - 109.9	≥ 110.0
Number										
Whole	21,710	65,154	127,678	176,129	198,652	146,733	84,787	41,856	24,804	5,075
Men	2,412	13,820	43,323	86,163	124,197	98,267	60,064	29,880	17,534	3,543
Women	19,298	51,334	84,355	89,966	74,455	48,466	24,723	11,976	7,270	1,532
Percentage of BS (%)										
Whole	12.5	13.5	14.6	15.4	15.9	16.8	18.3	20.2	22.1	25.4
Men	23.8	23.3	22.2	20.3	19.2	19.7	21.1	23.0	24.9	27.6
Women	11.1	10.9	10.7	10.6	10.3	10.9	11.6	13.3	15.4	20.2
Crude Odds ratio										
Whole	0.76*** (0.73 – 0.79)	0.83*** (0.81 - 0.85)	0.91*** (0.89 - 0.92)	0.96*** (0.95 - 0.98)	1	1.07 (1.05 – 1.09)	1.19*** (1.16 - 1.21)	1.34*** (1.31 - 1.38)	1.50*** (1.45 - 1.55)	1.80*** (1.69 - 1.92)
Men	1.31*** (1.19 – 1.44)	1.27*** (1.22 - 1.33)	1.20*** (1.17 - 1.23)	1.07*** (1.05 - 1.10)	1	1.03** (1.01- 1.05)	1.12** (1.09 - 1.15)	1.26*** (1.22 - 1.30)	1.39*** (1.34 - 1.45)	1.60*** (1.49 - 1.73)
Women	1.09** (1.03 – 1.14))	1.06** (1.02 - 1.10)	1.04* (1.01 - 1.07)	1.03* (1.00 - 1.07)	1	1.07*** (1.03 - 1.11)	1.14*** (1.09 - 1.20)	1.33*** (1.25 - 1.41)	1.57*** (1.47 - 1.69)	2.20*** (1.94 - 2.50)
Adjusted Odds ratio										
Whole	1.02 (0.98 – 1.07)	0.96** (0.94 – 0.99)	1.00 (0.97 - 1.02)	1.01 (0.99 - 1.03)	1	1.05*** (1.03 - 1.07)	1.10*** (1.08 - 1.13)	1.17*** (1.14 - 1.21)	1.23*** (1.19 - 1.27)	1.34*** (1.25 - 1.44)
Men	1.23*** (1.11 – 1.36)	1.11*** (1.07 – 1.17)	1.09*** (1.06 - 1.13)	1.03* (1.00 - 1.05)	1	1.04*** (1.02 - 1.06)	1.10*** (1.07 - 1.12)	1.16*** (1.12 - 1.19)	1.18*** (1.14 - 1.23)	1.21*** (1.12 – 1.32)
Women	1.08** (1.02 – 1.14)	0.99 (0.95 - 1.02)	1	1.06*** (1.03 - 1.09)	1.09*** (1.06 - 1.13)	1.20*** (1.15 - 1.24)	1.24*** (1.19 - 1.31)	1.36*** (1.28 – 1.45)	1.49*** (1.38 - 1.60)	1.79*** (1.56 – 2.06)

*P < 0.05, **P < 0.01, ***P < 0.001

Adjusted Odds ratios were calculated by controlling for age, smoking, pharmacotherapy for hypertension, pharmacotherapy for dyslipidemia, history of cardiovascular disease, moderate to heavy consumption of alcohol (≥ 46 g ethanol per day), habitual exercise, LND (late night dinner), LNS (late night snack), and sex (in the case of all subjects).

BS, breakfast skipping; WC, waist circumference.
